# Myocardial infarction with non-obstructive coronary arteries (MINOCA)

**DOI:** 10.3389/fcvm.2022.1032436

**Published:** 2022-11-15

**Authors:** Mehmet Yildiz, Namrita Ashokprabhu, Aarushi Shewale, Madison Pico, Timothy D. Henry, Odayme Quesada

**Affiliations:** ^1^The Carl and Edyth Lindner Center for Research and Education at the Christ Hospital, Cincinnati, OH, United States; ^2^Women’s Heart Center, The Christ Hospital Heart and Vascular Institute, Cincinnati, OH, United States

**Keywords:** myocardial infarction with non-obstructive coronary arteries, MINOCA, acute myocardial infarction, sex differences, coronary artery disease

## Abstract

Myocardial infarction with non-obstructive coronary arteries (MINOCA) is evident in up to 15% of all acute myocardial infarctions (AMI) and disproportionally affects females. Despite younger age, female predominance, and fewer cardiovascular risk factors, MINOCA patients have a worse prognosis than patients without cardiovascular disease and a similar prognosis compared to patients with MI and obstructive coronary artery disease (CAD). MINOCA is a syndrome with a broad differential diagnosis that includes both ischemic [coronary artery plaque disruption, coronary vasospasm, coronary microvascular dysfunction, spontaneous coronary artery dissection (SCAD), and coronary embolism/thrombosis] and non-ischemic mechanisms (Takotsubo cardiomyopathy, myocarditis, and non-ischemic cardiomyopathy)—the latter called MINOCA mimickers. Therefore, a standardized approach that includes multimodality imaging, such as coronary intravascular imaging, cardiac magnetic resonance, and in selected cases, coronary reactivity testing, including provocation testing for coronary vasospasm, is necessary to determine underlying etiology and direct treatment. Herein, we review the prevalence, characteristics, prognosis, diagnosis, and treatment of MINOCA -a syndrome often overlooked.

## Definition

Myocardial infarction (MI) without significant obstructive coronary artery disease (CAD) has been observed for decades in patients presenting with MI without a culprit artery. However, the term myocardial infarction with non-obstructive coronary arteries (MINOCA) was first introduced in 2013 (1), and it was not until 2017 that the European Society of Cardiology (ESC) position paper on MINOCA introduced diagnostic criteria for MINOCA based on the third universal definition of MI as follows: the presence of positive cardiac biomarker with clinical evidence of infarction, absence of stenosis (≥50%) in any epicardial coronary arteries on coronary angiography, and lack of any alternative diagnosis for the index presentation (2). The underlying pathophysiologic mechanisms in MINOCA include: coronary artery plaque disruption, coronary vasospasm, coronary microvascular dysfunction, spontaneous coronary artery dissection (SCAD), coronary embolism/thrombosis, Takotsubo cardiomyopathy, myocarditis, and non-ischemic cardiomyopathy. In 2018, the universal definition of MI was updated to include only ischemic mechanisms associated with myocardial injury, thereby excluding non-ischemic mechanisms such as Takotsubo cardiomyopathy, myocarditis, and non-ischemic cardiomyopathy (3). Therefore, the American Heart Association (AHA) scientific statement in 2019 excluded non-ischemic mechanisms from the MINOCA definition and labeled them MINOCA mimickers (4).

## Materials and methods

Electronic searches in MEDLINE were conducted through Aug 1, 2022, utilizing the following terms: MINOCA, plaque disruption, coronary vasospasm, coronary microvascular dysfunction, SCAD, coronary embolism/thrombosis, Takotsubo cardiomyopathy, myocarditis, and non-ischemic cardiomyopathy. All relevant retrospective and prospective observational studies and randomized clinical trials were considered without applying systemic inclusion or exclusion criteria. In addition, the reference lists of all relevant articles and reviews were manually searched.

## Prevalence, clinical characteristics, and prognosis in MINOCA

### Prevalence and clinical characteristics

MINOCA is considered a heterogeneous working diagnosis with an estimated prevalence of anywhere from 3 to 15% among all acute myocardial infarctions (AMI) patients (5–12). This heterogeneity is partly due to significant differences in what conditions are included in the term MINOCA and which definition is used. In a pooled analysis of 23 studies, the prevalence of MINOCA was 8.1% among 806,851 consecutive AMI patients (12). MINOCA has been reported by large national registries worldwide, including the US, Japan, Poland, and Sweden, with the incidence of MINOCA ranging from 2.9 to 10.2% (6, 9, 13, 14). Compared to MI with obstructive CAD, MINOCA patients were younger, with a median age of ∼61 years (12), and MINOCA was more common in Black (6, 8, 15) and Hispanic (16) patients. Further, MINOCA patients were less likely to present with traditional cardiovascular risk factors, including hypertension, dyslipidemia, diabetes, and current smoking history (12).

### Prognosis in MINOCA

Given the low-risk profile and non-obstructive coronary arteries, MINOCA has historically been thought to be a benign disease process. However, compared to patients without known cardiovascular disease, MINOCA patients have consistently been reported to be at higher risk for cardiovascular events (7). For example, in a pooled analysis of three studies, in-hospital and 1-month all-cause death rates were significantly higher in MINOCA patients compared with people without known cardiovascular disease (adjusted OR: 25.4, 95% CI: 1.4–465, *p* = 0.04 and adjusted OR: 9.7, 95% CI: 1.6–58.7, *p* = 0.03, respectively) (12).

On the other hand, studies assessing the prognosis of MINOCA patients compared to those with obstructive CAD are conflicting due to differences in how MINOCA was defined (17). While some reported a worse prognosis in MI with obstructive CAD compared to MINOCA (6, 8), others reported a similar prognosis between patients with MI with obstructive CAD and MINOCA (5, 13, 16). For example, in a pooled analysis of fourteen studies, the unadjusted 1-year mortality rates were 3.4% in MINOCA patients, and 1-year reinfarction rates were 2.6% (12). Compared with obstructive CAD, MINOCA patients had significantly lower in-hospital and 1-year all-cause death rates (OR: 0.36, 95% CI: 0.2–0.5, *p* < 0.001 and OR: 0.60, 95% CI: 0.5–0.7, *P*<0.001, respectively) (12). Whereas, a large retrospective study using a nationwide administrative database in Japan (*n* = 137,678) following the AHA 2019 scientific statement divided MINOCA patients into two groups: “true MINOCA” (those with underlying ischemic mechanisms, *n* = 13,022) and “working diagnosis of MINOCA” (those with underlying both ischemic and non-ischemic mechanisms, *n* = 14,045). The study suggested that regardless of the underlying mechanisms, both MINOCA groups were significantly associated with an increased risk of in-hospital death similar to patients with obstructive CAD (adjusted HR: 1.34, 95% CI: 1.21–1.48) and (adjusted HR: 1.34, 95% CI: 1.25–1.44), respectively (9).

Life-threatening ventricular arrhythmia and sudden cardiac death are not uncommon in patients with MI with obstructive CAD (18, 19). Similarly, myocardial ischemia, reperfusion injury, or inflammation around the scar tissue in MINOCA patients was hypothesized to precipitate ventricular arrhythmia (20). However, there has been little data to date on the incidence and prognosis of ventricular arrhythmia in patients with MINOCA. For example, in a small retrospective study of MINOCA and MINOCA mimicker patients with normal LV ejection fraction (*n* = 131), 18 developed ventricular arrhythmias during the index hospitalization, but none had sudden cardiac death (21). On the other hand, in a prospective study of young MINOCA patients, only 4 out of 299 had a cardiac arrest at presentation and had an automatic implantable cardioverter-defibrillator (16). In addition, no previous research has investigated supraventricular arrhythmia in MINOCA patients. Therefore, future studies are warranted to determine the incidence and prognosis of arrhythmias in MINOCA patients.

## ST-elevation MINOCA compared with non-ST-elevation MINOCA

The majority of MINOCA patients (70–80%) present with non-ST-elevation myocardial infarction (NSTEMI), limiting our understanding of ST-elevation myocardial infarction (STEMI) in MINOCA patients (12). ST-segment elevation at presentation is a predictor of all-cause death in MINOCA (5). And patients with STEMI have been shown to have a higher risk of mortality than those with NSTEMI (22, 23). However, studies investigating STEMI MINOCA are quite limited. A single-center, retrospective study of STEMI patients undergoing coronary angiography in the UK (*n* = 2,521) reported that only 4.4% of the study population had MINOCA, based on the ESC 2017 criteria. All-cause mortality rates were 3.6% in 30-day and 4.5% at 1-year follow-up (24). More recently, a single-center prospective registry in Denmark (*n* = 4,793) reported that 11% of STEMI activations had normal (0% stenosis) or non-obstructive (1–49%) coronary arteries but only 6.5% (*n* = 310) had elevated cardiac troponin. In a multivariable analysis, long-term mortality risk was twofold higher in patients with normal coronary arteries and elevated cardiac troponin than those with obstructive CAD (HR: 2.65, 95% CI: 1.52–4.61, *p* = 0.001) (25). To our knowledge, no studies to date have evaluated the difference in pathophysiologic mechanisms in NSTEMI vs. STEMI MINOCA, emphasizing the need for further research.

## The role of sex in MINOCA

MINOCA disproportionately affects females (26). Recent meta-analyses demonstrate that females comprise up to 50% of MINOCA patients (11, 12). Analysis of young patients with AMI in the VIRGO study (Variation in Recovery: Role of Gender on Outcomes of Young AMI Patients) showed that females had about five times higher odds of having MINOCA than males (14.9 vs. 3.5%, OR: 4.84, 95% CI: 3.29–7.13) (16). In the same study, 269 females had MINOCA, and the majority had MINOCA undefined (75%), 4% spasm, 21% dissection, and 1% embolization (16). The Heart Attack Research Program-Imaging Study (HARP), a study of females presenting with MINOCA primarily composed of NSTEMI, reported that plaque disruption was the most common cause of MINOCA (27).

Given the limited data, it remains unclear whether sex disparities in obstructive CAD are also evident in MINOCA. For example, in a small, single-center, prospective study of MINOCA patients, females were older and more likely to have a higher cardiovascular risk profile. However, the long-term prognosis was comparable in males and females (28). On the other hand, an analysis of MINOCA patients from ACTION Registry-GWTG (Acute Coronary Treatment and Intervention Outcomes Network Registry-Get With the Guidelines) showed a higher incidence of in-hospital MACE (a composite of death, reinfarction, cardiogenic shock, or heart failure) in females than males (5.4 vs. 4.1%; *p* < 0.0001) (8).

Although data on sex differences in MINOCA patients are limited, several studies have explored it in subtypes of MINOCA and MINOCA mimicker, i.e., SCAD and Takotsubo cardiomyopathy. SCAD disproportionately affects females (∼90%), particularly young females (29, 30). Although the underlying biological basis remains determined, pregnancy and hormonal replacement therapy are associated with a higher risk of SCAD in females (31). Pregnancy-associated SCAD is the most common cause of MI during pregnancy (32) and occurs predominantly during the third trimester and early postpartum (33). Notably, females with pregnancy-associated SCAD were more likely to present with high-risk cardiovascular features at presentation than females with SCAD outside of pregnancy (34). Sex disparities in SCAD, however, are poorly described. In a single-center, prospective cohort study of patients with SCAD (*n* = 288), only 8.7% were males; compared to females, males were younger, more likely to perform isometric exercise preceding SCAD, and less likely to have emotional stressors (35). There was no sex difference in long-term cardiovascular events (35). In terms of angiographic characteristics, a single-center, retrospective observational study reported that males were more likely to have type 1 SCAD and intimal tear than females (36).

Similarly, Takotsubo cardiomyopathy disproportionally affects females, particularly postmenopausal females, (37) sex differences have been reported. For instance, males are younger (38) and more likely to have physical stressors preceding Takotsubo cardiomyopathy (39), whereas females are older (38) and more likely to have emotional stressors preceding Takotsubo cardiomyopathy (39). In addition, the male sex was an independent predictor of short and long-term mortality risks in patients with Takotsubo cardiomyopathy (40, 41). In accordance with prior studies, a recent large multicenter registry-based cohort of patients with Takotsubo cardiomyopathy (*n* = 2,492), including 11% of males, reported males were younger but had a high prevalence of cardiovascular risk factors. In multivariable adjustment, the male sex increased in-hospital mortality risk by more than twofold and long-term mortality risk by more than 80% (42).

## Underlying pathophysiologic mechanisms in MINOCA

**FIGURE 1:**
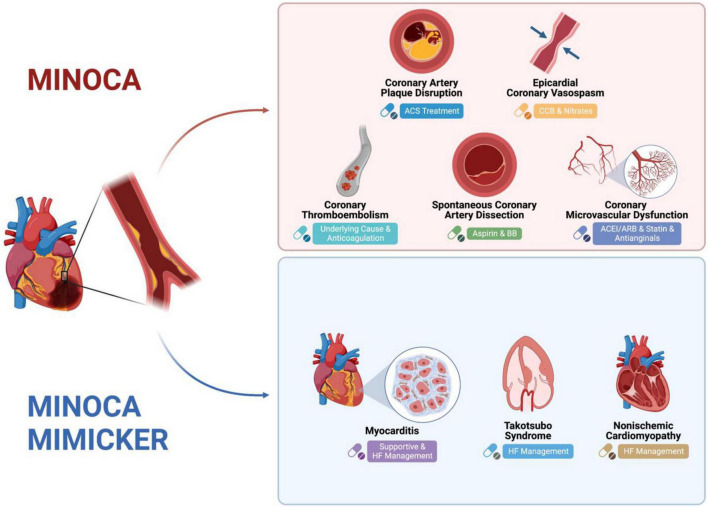
Mechanism of MINOCA and MINOCA-MIMICKERS and Differences in Treatment. Created with BioRender.com.

The underlying pathophysiologic mechanisms in MINOCA include: coronary artery plaque disruption, coronary vasospasm, SCAD, coronary embolism/thrombosis, MINOCA-mimickers (Takotsubo cardiomyopathy, myocarditis, and non-ischemic cardiomyopathy), and coronary microvascular dysfunction (Figure 1).

### Plaque disruption

Plaque disruption is an umbrella term that includes plaque rupture, erosion, and calcified nodules. As lipids accumulate in coronary arteries, the surge in inflammation, necrosis, fibrosis, and calcification leads to plaque formation, which may progress and be complicated by disruption (43). Plaque rupture results in the exposure of the plaque to the coronary lumen, which results in thrombosis and thromboembolism (44), while plaque erosion results from thrombus formation adjacent to the luminal surface following endothelial cell apoptosis and neutrophils’ recruitment without rupture (45). Female sex and smoking history are associated with an increased risk of plaque erosion compared to rupture (46). Plaque rupture can be detected utilizing intravascular imaging including intravascular angiography ultrasound (IVUS) or coronary optical coherence tomography (OCT); whereas the higher resolution of OCT is needed to assess plaque erosion (47). In a multi-center prospective study, plaque rupture (and ulceration) existed in 38% of females with MINOCA (*n* = 16/42) who underwent IVUS (48). The HARP study reported that plaque disruption was the most common cause of MINOCA, as was evident in 43.4% of females with MINOCA who underwent OCT including 8 with plaque rupture, 5 with plaque erosion, and the rest had an intra-plaque cavity or layered plaque (27). Notably, only 59% of participants had three-vessel OCT, which may have led to an underestimation of the prevalence of plaque disruption (27).

### Coronary vasospasm

Coronary vasospasm is defined as reproducible nitrate-responsive chest pain with transient ischemic EKG changes and >90% vasoconstriction on angiography in provocative testing with acetylcholine or ergonovine (49). The predominant pathophysiological mechanism is hyper-reactivity within the vascular smooth muscle in either the epicardial or microvascular vessels (50). Coronary vasospasm is a common cause of MINOCA, with about half of MINOCA patients having a positive provocative test in a single-center prospective study (51). Asians are at increased risk of coronary vasospasm with up to threefold higher risk of acetylcholine-provoked coronary vasospasm than Whites (52). Smoking is a known risk factor for coronary vasospasm (53). In contrast, other traditional risk factors such as sex, hypertension, diabetes mellitus, or hyperlipidemia were unrelated to increased coronary vasospasm risk (54). Coronary vasospasm is associated with a 2.5–13% long-term risk of MACE (55).

### Spontaneous coronary artery dissection

SCAD results from the formation of a false lumen within the wall of epicardial coronary arteries in the absence of atherosclerosis (31). Two potential pathological mechanisms have been described, including an inside-out model where a tear in the intima layer resulting in a false lumen and an outside-in model where the formation of a false lumen following a spontaneous intramural hemorrhage with or without an intimal tear (31). Based on angiographic characteristics, four types of SCADs have been described: type 1 describes multiple radiolucent lumens and staining within the arterial wall, type 2 describes diffuse and smooth stenosis, type 3 describes focal stenosis mimicking atherosclerosis, and type 4 describes total vessel occlusion (29, 30). Although coronary angiography has limitations, it remains the principal diagnostic tool for SCAD (29, 30). In cases of diagnostic uncertainty or need for any invasive intervention, advanced intracoronary imaging modalities, IVUS or OCT, may be considered with caution, given the risk of propagating the dissection (29, 30).

The true prevalence of SCAD is unknown, given that the diagnosis is often missed. In the largest study of STEMI, SCAD was evident in only 1% of STEMI patients and was more prevalent in females (93%) (56). SCAD that results in a non-obstructive lesion or is missed at initial presentation is considered MINOCA as recommended by the AHA statement, whereas SCAD that results in complete vessel occlusion (type 4) is excluded (4). The predisposing mechanisms for SCAD are multifactorial, including genetic factors, fibromuscular dysplasia, pregnancy, female sex hormones, systemic inflammatory condition, and precipitating factors such as emotional stress, rigorous physical activity, or stimulant drugs (29, 30).

### Coronary embolism/thrombosis

Coronary embolism is often underdiagnosed and understudied. The type of coronary embolism is dependent on the thrombus origin: direct (from the left atrium, left ventricle, or valvular origin), paradoxical (from the venous circulation to systemic circulation through septal defects), or iatrogenic (from intracoronary or valvular interventions) (57). A retrospective analysis of a national database in Japan revealed that only 2.9% of all AMI patients had a coronary embolism, of which 73% were secondary to atrial fibrillation (58). During a median follow-up of 49 months, 1 out of 10 patients with coronary embolism developed recurrent thromboembolic episodes despite most patients having a CHADS2 score of 0 or 1 (58). Certain conditions, such as hereditary or acquired thrombophilia, increase the risk of coronary thromboembolism. In a meta-analysis of eight studies of MINOCA with available thrombophilia screening data, 14% had hereditary thrombophilia, with Factor V Leiden as the most common (11). Therefore, an extensive evaluation, including hypercoagulable workup, monitoring for atrial fibrillation, and assessment for patent foramen ovale, is needed to determine the underlying causes of coronary embolism (59).

### MINOCA mimickers

Takotsubo cardiomyopathy, also commonly referred to as stress-induced cardiomyopathy or broken heart syndrome, is characterized by reversible wall motion abnormalities without obstructive CAD due to a surge of catecholamines (60). Four major anatomical variants are observed: apical ballooning (most common), mid-left ventricular, basal, and biventricular (61). Clinical presentations vary but often follow emotional or physical stress, such as critical illness (62). Takotsubo cardiomyopathy is more prevalent in females, especially those postmenopausal females (63). Several studies suggested that coronary artery vasospasm and microvascular dysfunction may be part of the pathophysiologic mechanism (64). Diagnosis is often made with coronary angiography demonstrating an absence of obstructive CAD and characteristic wall motion abnormality on the left ventriculogram and evidence of complete recovery of left ventricular function and wall motion abnormality on followed-up transthoracic echocardiogram (62). Cardiac magnetic resonance imaging (CMRI) is a useful tool in Takotsubo cardiomyopathy to exclude other causes of AMI (63). Although Takotsubo cardiomyopathy is reversible, cardiogenic shock and death risks are comparable to AMI patients with CAD in several observational cohorts (65–67).

Myocarditis or inflammatory cardiomyopathy is commonly caused by viral infections, but it can also be caused by bacterial infections, toxic substances, or autoimmune disorders (68). Myocarditis is more common in younger patients, although it affects patients of all ages. Fulminant myocarditis, although rare, can result in life-threatening cardiogenic shock (69). Diagnosis of myocarditis is made using CMRI characterized by the presence of diffuse myocardial edema on T2 and with myocardial biopsy (70, 71). In a meta-analysis of five observational studies with available CMRI data, one-third of MINOCA patients had myocarditis. It was more common in younger patients and those with high C-reactive protein (72).

Non-ischemic cardiomyopathy includes dilated cardiomyopathy (most common), hypertrophic cardiomy-opathy, restrictive cardiomyopathy, and arrhythmogenic cardiomyopathy (73). A longitudinal observational study assessing the prognostic role of CMRI in MINOCA patients revealed that 25% of the participants had MINOCA due to non-ischemic cardiomyopathy. Further, non-ischemic cardiomyopathy was associated with the highest mortality compared to other mechanisms of MINOCA (74). Using stress CMRI, underlying microvascular dysfunction has been reported in patients with dilated cardiomyopathy (75).

### Coronary microvascular dysfunction

It remains debatable whether microvascular dysfunction is among the causes of MINOCA, given the lack of data to date. Coronary microvascular dysfunction stems from impaired vasodilation, increased vasoconstriction, and abnormal remodeling of microcirculation, which alters the coronary flow reserve (CFR) in the absence of epicardial obstructive disease (76). Coronary microvascular dysfunction is often underdiagnosed because it requires invasive functional tests (77). The majority of studies assessing coronary microvascular dysfunction have been completed among patients with ischemia with no obstructive coronary arteries (INOCA), with a prevalence of up to 41% (78). However, in a small prospective study of MINOCA patients (*n* = 40) who underwent stress CMRI, 25% had low myocardial perfusion reserve index (≤1.84), without evidence of late gadolinium enhancement on CMRI or plaque disruption on IVUS (79). Further research is needed to determine if microvascular dysfunction is one of the causes of MINOCA.

## Diagnostic modalities for MINOCA

MINOCA is a syndrome, not a diagnosis, requiring a comprehensive diagnostic workup. Coronary angiography is the first-line diagnostic tool to detect non-obstructive epicardial coronary arteries (<50% stenosis) in the setting of an MI. Although its role is limited to identifying the underlying mechanisms, it may offer leading insights; such as evidence of haziness or filling defect in plaque disruption, fresh thrombosis with the reduced distal flow in coronary embolism/thrombosis, evidence of false lumen in SCAD, and evidence of apical ballooning on the left ventricular angiogram in Takotsubo cardiomyopathy (4). Therefore, advanced imaging modalities are vital in diagnosing and identifying the underlying mechanisms of MINOCA (80).

### Coronary intravascular imaging

Coronary intravascular imaging with IVUS and OCT is essential in evaluating MINOCA to diagnose plaque disruption. It should be performed at the time of coronary angiography for AMI in all 3 major epicardial arteries. In MINOCA patients, IVUS detected plaque disruption in up to 40% of cases (48) and OCT detected the underlying culprit lesion in about half of cases (27). Intravascular imaging can also be useful in the evaluation of SCAD in selected cases where diagnosis in uncertain.

### Cardiac imaging

Transthoracic echocardiography is useful in the assessment of cardiac function after a MINOCA event. It can be used in the diagnosis of takotsubo cardiomyopathy and non-ischemic cardiomyopathy and is particularly helpful in follow-up to demonstrate recovery of left ventricular function (81). In addition, transesophageal echocardiography might be considered in the workup when coronary embolism is suspected (82).

CMRI is recommended by the ESC guidelines in all MINOCA cases where a diagnosis is uncertain (2). CMRI provides an appropriate diagnosis in 74–87% of all MINOCA patients (74, 83–87). CMRI subendocardial (or transmural) pattern of myocardial edema, inflammation, or fibrosis is suggestive of ischemic MI, whereas an epicardial pattern is suggestive of non-ischemic MI (81). Native T1 and extracellular volume mapping detect areas of fibrosis and edema that, in a vascular pattern, are suggestive of MI even in the absence of late gadolinium enhancement. Further, CMRI, like an echocardiogram, can be used to diagnose takotsubo cardiomyopathy and non-ischemic cardiomyopathy, but CMRI is the only imaging modality that can be used to diagnose myocarditis. In addition, myocardial perfusion quantification with adenosine or regadenoson can be used to diagnose coronary microvascular dysfunction non-invasively (88). Newer technologies such as high-resolution late gadolinium enhancement CMRI, using a 3-dimensional respiratory-navigated method, modified about half of the uncertain diagnoses (48%) in MINOCA patients, where transthoracic echocardiography, ventriculography, and conventional CMRI were non-diagnostic (89).

The timing of CMRI from the time of the event is critically important, and CMRI is recommended to be completed as close to the AMI as possible. The diagnostic performance of CMRI increased significantly from 47% when performed at a median of 12 days to 77% when performed at a median of 3 days after hospital admission (87). CMRI also carries not only diagnostic but also prognostic value. CMRI confirmed MI was an independent predictor of long-term cardiovascular events (86).

### Invasive functional studies

Provocative spasm testing using acetylcholine or ergonovine (no longer available in the US) is used to diagnose coronary epicardial or microvascular vasospasm and endothelial-dependent coronary microvascular dysfunction (90). Provocative spasm testing is performed with the administration of intracoronary acetylcholine. A recent meta-analysis of studies assessing procedural complications of intracoronary acetylcholine was encouraging as the overall incidence of complications following intracoronary acetylcholine was only 0.5%, without any incidence of death (91). Moreover, there was no significant change in safety and diagnostic yield between administrating 100 vs. 200 μg of intracoronary acetylcholine (91). A recent study on the use of provocative testing at the initial presentation of MINOCA patients reported no complications (51). They demonstrated that provocative spasm testing in MINOCA cases suspicious for vasospasm was positive in about half of the patients (46.2%) with 64.9% having evidence of epicardial spasm and 35.1% microvascular spasm (51). Patients with coronary vasospasm (epicardial or microvascular) had a significantly higher risk of cardiac death than those without coronary spasm at a median 3-year follow-up (51).

CFR is assessed by the thermodilution or doppler flow velocity method in a hyperemic state using adenosine. CFR <2.0 is used to diagnose non-endothelial-dependent coronary microvascular dysfunction (77). Index of microvascular resistance (IMR) is another useful technique to assess coronary microvascular dysfunction by thermodilution with >25 used as the cut-off but is associated with lower sensitivity and specificity than CFR. Although coronary microvascular dysfunction is associated with an increased risk of MACE in INOCA patients (92), its prognostic impact on MINOCA patients is less clear. However, a recent meta-analysis demonstrated that a low CFR was predictive of mortality not only in patients with acute and chronic coronary syndromes but also in patients with heart failure, aortic stenosis, and systemic sclerosis (93).

### Multimodality approach

**FIGURE 2:**
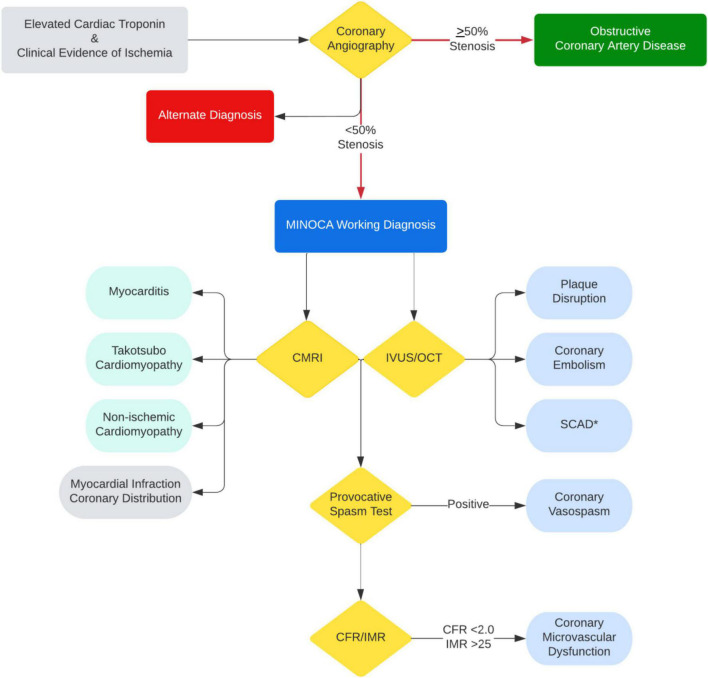
Diagnostic algorithm with multimodality imaging and testing for MINOCA and MINOCA mimicker. *IVUS/OCT should be avoided if SCAD is suspected unless invasive treatment is planned due to ongoing ischemia.

Multimodality imaging, both invasive and non-invasive, is critical to determining the underlying diagnosis of MINOCA. As outlined above, each modality has its limitations in reaching the underlying diagnosis; however, the diagnostic yield significantly improves when coupled (94, 95). The HARPP study demonstrated the combination of OCT and CMRI resulted in a diagnosis in 85% of the cases whereas OCT alone was diagnostic in 46% and CMRI alone in 74% of cases (27). Therefore, in a timely manner, CMRI needs to be performed for all patients with suspected MINOCA where a diagnosis remains uncertain. Further, IVUS or OCT of all three vessels is warranted at the time of angiography to identify intracoronary mechanisms leading to MINOCA. And in appropriate cases, provocative spasm testing with acetylcholine and measurement of CFR and/or IMR should be considered (Figure 2).

## Treatment

The management strategies for MINOCA should be tailored to the underlying diagnosis (96). For example, aspirin and high-intensity statin should be used in patients with plaque disruption. In addition, those with plaque disruption not undergoing stenting may be treated with dual antiplatelet therapy by adding ticagrelor for ≤1 month, based on the low revascularization rates at 1- and 4-year follow-up, 5.7 and 21.1%, respectively (97). Beta-blocker and renin-angiotensin system inhibitors should be considered in those with left ventricular dysfunction (4). Long-acting calcium channel antagonists (dihydropyridine and non-dihydropyridine) are used widely in MINOCA patients secondary to epicardial coronary vasospasm given that mechanistically they relax vascular smooth muscles secondary to suppressed Ca^2+^ flow (4). In cases of refractory variant angina, nitrates can be added to calcium channel antagonists, which enables vascular smooth muscle relaxation through nitric oxide reduction (98). Coronary embolism or thrombosis are treated with antithrombotic agents and targeted therapies for underlying thrombophilia (99).

Based on non-randomized observations, conservative management is favored in patients with SCAD over percutaneous coronary intervention (PCI), given that majority of dissections heal with conservative management and the increased risk of complications with intervention (30). PCI should be reserved for those with STEMI, cardiogenic shock, or ongoing ischemia. The use of antithrombotic agents in SCAD is controversial during the acute event (100). Secondary prevention medications in SCAD, such as aspirin, beta-blocker, statin, and renin-angiotensin system inhibitors, should be assessed based on the individual’s risk factors (29). Data on treating coronary microvascular dysfunction is limited and derived from patients with INOCA, where statins and renin-angiotensin system inhibitors have been shown to improve CFR (101). Antianginal treatment with B-blockers, calcium channel antagonists, and ranolazine for patients with chest pain is utilized (101).

Management of MINOCA mimickers is predominantly supportive care and guideline directed medical therapy for heart failure although data on this is limited. Majority of patients with Takotsubo cardiomyopathy have spontaneous recovery of normal cardiac function (63). However, those with left ventricular dysfunction are treated with beta-blockers and renin-angiotensin system inhibitors, and those with progressive circulatory failure may require mechanical circulatory support (102). Myocarditis often resolves in most patients within 2–4 weeks. However, in patients that develop arrhythmia and persistent cardiac dysfunction guideline-directed medical therapy (69). Physical activity should be avoided in the acute phase and up to 6 months (69). There are ongoing trials assessing targeted treatment options for underlying etiologies, including anti-virals and immunosuppressives, that will provide better insight into targeted therapies in the future (68).

Secondary prevention medications are less frequently utilized in MINOCA patients than in those with obstructive CAD (103–106). A large multi-center, registry-based cohort study of patients with CAD undergoing cardiac catheterization (*n* = 1,489,745) reported that prescriptions of aspirin, statin, B-blocker, and renin-angiotensin system inhibitors were significantly lower in patients with non-obstructive coronary arteries than those with obstructive CAD (*p* < 0.0001 for each) (104). Not only at discharge but even at 1-year follow-up, secondary prevention medications were used substantially lower in patients with non-obstructive than those with obstructive CAD, as reported in a *post hoc* analysis of the multicenter prospective registry (105).

Studies have demonstrated significant benefits of secondary prevention in MINOCA patients. For example, in an extensive nationwide registry of patients with AMI in Sweden, MINOCA patients discharged on statins and renin-angiotensin system inhibitors experienced 23 and 18% lower risks of any MACE during a mean follow-up of over 4 years (14). Likewise, a Korean national registry reported that the lack of statins and renin-angiotensin system inhibitors at discharge in MINOCA patients increased the risk of 2-year all-cause of death by more than twofold in a multivariable-adjusted analysis (5). In addition, a meta-analysis of five observational studies reported 35% reduced mortality risk with statin treatment in MINOCA patients (HR: 0.65; 95% CI: 0.56–0.75) (107). Antiplatelet therapy is a cornerstone in managing obstructive coronary arteries; however, its role is less clear in MINOCA patients. For example, there was a trend toward increased risk of harm with aspirin in a small retrospective analysis (108), and with clopidogrel in a *post hoc* analysis (109).

Management of MINOCA is based on retrospective analyses with several limitations and potential biases. Therefore, it is essential to design randomized control trials to improve outcomes in MINOCA patients. In that respect, two randomized control trials are underway; NCT03686696 investigates the benefit of renin-angiotensin system inhibitors and B-blockers with a 2:2 factorial design in MINOCA patients and NCT04538924 compares two treatment groups (statin, renin-angiotensin system inhibitor, B-blocker, and dual-antiplatelet therapy vs. statin and renin-angiotensin system inhibitor) with a 1:1 ratio in MINOCA patients (110, 111). Further, a randomized PROMISE trial, NCT05122780, is underway, assessing precision medicine vs. standard of care for the underlying cause of MINOCA (112).

## Conclusion

MINOCA is a syndrome that predominately affects females with a heterogenous working diagnosis that is understudied, underdiagnosed, and undertreated. MINOCA has similar prognosis compared to patients with MI and obstructive CAD. Therefore, diagnostic workup that includes multimodality advanced imaging, is essential to identify the underlying mechanisms and guide treatment. Randomized clinical trials on secondary prevention are underway but further research is needed to guide specific treatment of underlying mechanism.

## Data availability statement

The original contributions presented in this study are included in the article/supplementary material, further inquiries can be directed to the corresponding author.

## Ethics statement

Ethical review and approval was not required for this study in accordance with the local legislation and institutional requirements. Written informed consent was not required for this study in accordance with the local legislation and institutional requirements.

## Author contributions

MY, NA, AS, and MP reviewed the literature, wrote, and edited the manuscript. OQ and TH created outline for the manuscript, wrote, revised, and edited the manuscript. All authors contributed to the article and approved the submitted version.

## Abbreviations

AMI: Acute myocardial infarction
CAD: Coronary artery disease
CFR: Coronary flow reserve
CMRI: Cardiac magnetic resonance imaging
IMR: Index of microvascular resistance
INOCA: Ischemia with no obstructive coronary arteries
IVUS: Intravascular angiography ultrasound
MI: Myocardial infarction
MINOCA: Myocardial infarction with non-obstructive coronary arteries
NSTEMI: Non-ST-elevation myocardial infarction
OCT: Optical coherence tomography
SCAD: Spontaneous coronary artery dissection
STEMI: ST-elevation myocardial infarction.

## References

[1] Beltrame JF. Assessing patients with myocardial infarction and nonobstructed coronary arteries (MINOCA). *J Int Med.* (2013) 273:182–5. 10.1111/j.1365-2796.2012.02591.x 22998397

[2] Agewall S Beltrame JF Reynolds HR Niessner A Rosano G Caforio ALP ESC working group position paper on myocardial infarction with non-obstructive coronary arteries. *Eur Heart J.* (2016) 38:ehw149. 10.1093/eurheartj/ehw149 28158518

[3] Thygesen K Alpert JS Jaffe AS Chaitman BR Bax JJ Morrow DA Fourth universal definition of myocardial infarction (2018). *Eur Heart J.* (2019) 40:237–69. 10.1093/eurheartj/ehy462 30165617

[4] Tamis-Holland JE Jneid H Reynolds HR Agewall S Brilakis ES Brown TM Contemporary diagnosis and management of patients with myocardial infarction in the absence of obstructive coronary artery disease: a scientific statement from the american heart association. *Circulation.* (2019) 139:e891–908. 10.1161/CIR.0000000000000670 30913893

[5] Choo EH Chang K Lee KY Lee D Kim JG Ahn Y Prognosis and predictors of mortality in patients suffering myocardial infarction with non-obstructive coronary arteries. *J Am Heart Assoc.* (2019) 8:e011990. 10.1161/JAHA.119.011990 31284804 PMC6662150

[6] Dreyer RP Tavella R Curtis JP Wang Y Pauspathy S Messenger J Myocardial infarction with non-obstructive coronary arteries as compared with myocardial infarction and obstructive coronary disease: outcomes in a medicare population. *Eur Heart J.* (2020) 41:870–8. 10.1093/eurheartj/ehz403 31222249 PMC7778433

[7] Eggers KM Hjort M Baron T Jernberg T Nordenskjöld AM Tornvall P Morbidity and cause-specific mortality in first-time myocardial infarction with nonobstructive coronary arteries. *J Intern Med.* (2019) 285:419–28. 10.1111/joim.12857 30474313

[8] Smilowitz NR Mahajan AM Roe MT Hellkamp AS Chiswell K Gulati M Mortality of myocardial infarction by sex, age, and obstructive coronary artery disease status in the ACTION registry–GWTG (acute coronary treatment and intervention outcomes network registry–get with the guidelines). *Circ Cardiovasc Qual Outcomes.* (2017) 10:e003443. 10.1161/CIRCOUTCOMES.116.003443 29246884

[9] Ishii M Kaikita K Sakamoto K Seki T Kawakami K Nakai M Characteristics and in-hospital mortality of patients with myocardial infarction in the absence of obstructive coronary artery disease in super-aging society. *Int J Cardiol.* (2020) 301:108–13. 10.1016/j.ijcard.2019.09.037 31740139

[10] Bainey KR Welsh RC Alemayehu W Westerhout CM Traboulsi D Anderson T Population-level incidence and outcomes of myocardial infarction with non-obstructive coronary arteries (MINOCA): insights from the alberta contemporary acute coronary syndrome patients invasive treatment strategies (COAPT) study. *Int J Cardiol.* (2018) 264:12–7. 10.1016/j.ijcard.2018.04.004 29655952

[11] Pasupathy S Air T Dreyer RP Tavella R Beltrame JF. Systematic review of patients presenting with suspected myocardial infarction and nonobstructive coronary arteries. *Circulation.* (2015) 131:861–70. 10.1161/CIRCULATIONAHA.114.011201 25587100

[12] Pasupathy S Lindahl B Litwin P Tavella R Williams MJA Air T Survival in patients with suspected myocardial infarction with nonobstructive coronary arteries: a comprehensive systematic review and meta-analysis from the MINOCA global collaboration. *Circ Cardiovasc Qual Outcomes.* (2021) 14:e007880. 10.1161/CIRCOUTCOMES.121.007880 34784229

[13] Gasior P Desperak A Gierlotka M Milewski K Wita K Kalarus Z Clinical characteristics, treatments, and outcomes of patients with myocardial infarction with non-obstructive coronary arteries (MINOCA): results from a multicenter national registry. *J Clin Med.* (2020) 9:2779. 10.3390/jcm9092779 32867273 PMC7564426

[14] Lindahl B Baron T Erlinge D Hadziosmanovic N Nordenskjöld A Gard A Medical therapy for secondary prevention and long-term outcome in patients with myocardial infarction with nonobstructive coronary artery disease. *Circulation.* (2017) 135:1481–9. 10.1161/CIRCULATIONAHA.116.026336 28179398

[15] Larsen AI Nilsen DWT Yu J Mehran R Nikolsky E Lansky AJ Long-term prognosis of patients presenting with st-segment elevation myocardial infarction with no significant coronary artery disease (from the HORIZONS-AMI trial). *Am J Cardiol.* (2013) 111:643–8. 10.1016/j.amjcard.2012.11.011 23261001

[16] Safdar B Spatz ES Dreyer RP Beltrame JF Lichtman JH Spertus JA Presentation, clinical profile, and prognosis of young patients with myocardial infarction with nonobstructive coronary arteries (MINOCA): results from the VIRGO study. *J Am Heart Assoc.* (2018) 7:9174. 10.1161/JAHA.118.009174 29954744 PMC6064896

[17] Niccoli G Camici PG. Myocardial infarction with non-obstructive coronary arteries: what is the prognosis? *Eur Heart J Suppl.* (2020) 22:E40–5. 10.1093/eurheartj/suaa057 32523437 PMC7270909

[18] Al-Khatib SM Stevenson WG Ackerman MJ Bryant WJ Callans DJ Curtis AB 2017 AHA/ACC/HRS guideline for management of patients with ventricular arrhythmias and the prevention of sudden cardiac death. *Circulation.* (2018) 138:e272–391. 10.1161/CIR.0000000000000549 29084731

[19] Zeppenfeld K Tfelt-Hansen J de Riva M Winkel BG Behr ER Blom NA 2022 ESC guidelines for the management of patients with ventricular arrhythmias and the prevention of sudden cardiac death. *Eur Heart J.* (2022) 2022:262. 10.1093/eurheartj/ehac262 36477551

[20] Kosmas N Manolis AS Dagres N Iliodromitis EK. Myocardial infarction or acute coronary syndrome with non-obstructive coronary arteries and sudden cardiac death: a missing connection. *EP Eur.* (2020) 22:1303–10. 10.1093/europace/euaa156 32894280 PMC7478321

[21] Bière L Niro M Pouliquen H Gourraud J-B Prunier F Furber A Risk of ventricular arrhythmia in patients with myocardial infarction and non-obstructive coronary arteries and normal ejection fraction. *World J Cardiol.* (2017) 9:268. 10.4330/wjc.v9.i3.268 28400924 PMC5368677

[22] Johnston N Jönelid B Christersson C Kero T Renlund H Schenck-Gustafsson K Effect of gender on patients with ST-elevation and non-ST-elevation myocardial infarction without obstructive coronary artery disease. *Am J Cardiol.* (2015) 115:1661–6. 10.1016/j.amjcard.2015.03.006 25900352

[23] Frycz-Kurek AM Gierlotka M Gąsior M Wilczek K Lekston A Kalarus Z Patients with no significant lesions in coronary arteries and ST-segment elevation myocardial infarction have worse outcome than patients with non-ST-segment elevation myocardial infarction: analysis from PL-ACS Registry. *Kardiol Pol.* (2010) 68:1211–7. 21108194

[24] Gue YX Corballis N Ryding A Kaski JC Gorog DA. MINOCA presenting with STEMI: incidence, aetiology and outcome in a contemporaneous cohort. *J Thromb Throm.* (2019) 48:533–8. 10.1007/s11239-019-01919-5 31327089

[25] Andersson HB Pedersen F Engstrøm T Helqvist S Jensen MK Jørgensen E Long-term survival and causes of death in patients with ST-elevation acute coronary syndrome without obstructive coronary artery disease. *Eur Heart J.* (2018) 39:102–10. 10.1093/eurheartj/ehx491 29029035

[26] Pacheco Claudio C Quesada O Pepine CJ Noel Bairey Merz C. Why names matter for women: MINOCA/INOCA (myocardial infarction/ischemia and no obstructive coronary artery disease). *Clin Cardiol.* (2018) 41:185–93. 10.1002/clc.22894 29498752 PMC6489859

[27] Reynolds HR Maehara A Kwong RY Sedlak T Saw J Smilowitz NR Coronary optical coherence tomography and cardiac magnetic resonance imaging to determine underlying causes of myocardial infarction with nonobstructive coronary arteries in women. *Circulation.* (2021) 143:624–40. 10.1161/CIRCULATIONAHA.120.052008 33191769 PMC8627695

[28] Gao S Ma W Huang S Lin X Yu M. Sex-specific clinical characteristics and long-term outcomes in patients with myocardial infarction with non-obstructive coronary arteries. *Front Cardiovasc Med.* (2021) 8:670401. 10.3389/fcvm.2021.670401 34179135 PMC8221425

[29] Hayes SN Kim ESH Saw J Adlam D Arslanian-Engoren C Economy KE Spontaneous coronary artery dissection: current state of the science: a scientific statement from the american heart association. *Circulation.* (2018) 137:e523–57. 10.1161/CIR.0000000000000564 29472380 PMC5957087

[30] Adlam D Alfonso F Maas A Vrints C Hussaini A Bueno H European society of cardiology, acute cardiovascular care association, SCAD study group: a position paper on spontaneous coronary artery dissection. *Eur Heart J.* (2018) 39:3353–68. 10.1093/eurheartj/ehy080 29481627 PMC6148526

[31] di Fusco SA Rossini R Zilio F Pollarolo L di Uccio FS Iorio A Spontaneous coronary artery dissection: overview of pathophysiology. *Trends Cardiovasc Med.* (2022) 32:92–100. 10.1016/j.tcm.2021.01.002 33453416

[32] Elkayam U Jalnapurkar S Barakkat MN Khatri N Kealey AJ Mehra A Pregnancy-associated acute myocardial infarction. *Circulation.* (2014) 129:1695–702. 10.1161/CIRCULATIONAHA.113.002054 24753549

[33] Faden MS Bottega N Benjamin A Brown RN. A nationwide evaluation of spontaneous coronary artery dissection in pregnancy and the puerperium. *Heart.* (2016) 102:1974–9. 10.1136/heartjnl-2016-309403 27411842

[34] Tweet MS Hayes SN Codsi E Gulati R Rose CH Best PJM. Spontaneous coronary artery dissection associated with pregnancy. *J Am Coll Cardiol.* (2017) 70:426–35. 10.1016/j.jacc.2017.05.055 28728686

[35] Fahmy P Prakash R Starovoytov A Boone R Saw J. Pre-disposing and precipitating factors in men with spontaneous coronary artery dissection. *JACC Cardiovasc Interv.* (2016) 9:866–8. 10.1016/j.jcin.2016.02.024 27101917

[36] Zilio F Muraglia S Morat F Borghesi M Todaro D Menotti A Sex differences in clinical and angiographic characteristics in spontaneous coronary artery dissection. *Future Cardiol.* (2021) 17:669–75. 10.2217/fca-2020-0124 33078958

[37] Omerovic E Citro R Bossone E Redfors B Backs J Bruns B Pathophysiology of takotsubo syndrome – a joint scientific statement from the heart failure association takotsubo syndrome study group and myocardial function working group of the European society of cardiology – part 2: vascular pathophysiology, gender and sex hormones, genetics, chronic cardiovascular problems and clinical implications. *Eur J Heart Fail.* (2022) 24:274–86. 10.1002/ejhf.2368 34655287

[38] Cammann VL Szawan KA Stähli BE Kato K Budnik M Wischnewsky M Age-related variations in takotsubo syndrome. *J Am Coll Cardiol.* (2020) 75:1869–77. 10.1016/j.jacc.2020.02.057 32327096

[39] Agdamag AC Patel H Chandra S Rao A Suboc TM Marinescu K Sex differences in takotsubo syndrome: a narrative review. *J Womens Health.* (2020) 29:1122–30. 10.1089/jwh.2019.7741 31549884

[40] Santoro F Núñez Gil IJ Stiermaier T El-Battrawy I Guerra F Novo G Assessment of the german and italian stress cardiomyopathy score for risk stratification for in-hospital complications in patients with takotsubo syndrome. *JAMA Cardiol.* (2019) 4:892. 10.1001/jamacardio.2019.2597 31389988 PMC6686773

[41] Stiermaier T Moeller C Oehler K Desch S Graf T Eitel C Long-term excess mortality in takotsubo cardiomyopathy: predictors, causes and clinical consequences. *Eur J Heart Fail.* (2016) 18:650–6. 10.1002/ejhf.494 26990821

[42] Arcari L Núñez-Gil IJ Stiermaier T El-Battrawy I Guerra F Novo G Gender differences in takotsubo syndrome. *J Am Coll Cardiol.* (2022) 79:2085–93. 10.1016/j.jacc.2022.03.366 35618345 PMC8972425

[43] Bentzon JF Otsuka F Virmani R Falk E. Mechanisms of plaque formation and rupture. *Circ Res.* (2014) 114:1852–66. 10.1161/CIRCRESAHA.114.302721 24902970

[44] Virmani R Burke AP Farb A Kolodgie FD. Pathology of the vulnerable plaque. *J Am Coll Cardiol.* (2006) 47:C13–8. 10.1016/j.jacc.2005.10.065 16631505

[45] Quillard T Araújo HA Franck G Shvartz E Sukhova G Libby P. TLR2 and neutrophils potentiate endothelial stress, apoptosis and detachment: implications for superficial erosion. *Eur Heart J.* (2015) 36:1394–404. 10.1093/eurheartj/ehv044 25755115 PMC4458287

[46] White SJ Newby AC Johnson TW. Endothelial erosion of plaques as a substrate for coronary thrombosis. *Thromb Haemost.* (2016) 115:509–19. 10.1160/th15-09-0765 26791872

[47] Araki M Park S-J Dauerman HL Uemura S Kim J-S di Mario C Optical coherence tomography in coronary atherosclerosis assessment and intervention. *Nat Rev Cardiol.* (2022) 2022:687. 10.1038/s41569-022-00687-9 35449407 PMC9982688

[48] Reynolds HR Srichai MB Iqbal SN Slater JN Mancini GBJ Feit F Mechanisms of myocardial infarction in women without angiographically obstructive coronary artery disease. *Circulation.* (2011) 124:1414–25. 10.1161/CIRCULATIONAHA.111.026542 21900087 PMC3619391

[49] Beltrame JF Crea F Kaski JC Ogawa H Ong P Sechtem U International standardization of diagnostic criteria for vasospastic angina. *Eur Heart J.* (2015) 38:ehv351. 10.1093/eurheartj/ehv351 26245334

[50] Shimokawa H. 2014 williams harvey lecture: importance of coronary vasomotion abnormalities–from bench to bedside. *Eur Heart J.* (2014) 35:3180–93. 10.1093/eurheartj/ehu427 25354517

[51] Montone RA Niccoli G Fracassi F Russo M Gurgoglione F Cammà G Patients with acute myocardial infarction and non-obstructive coronary arteries: safety and prognostic relevance of invasive coronary provocative tests. *Eur Heart J.* (2017) 39:91–8. 10.1093/eurheartj/ehx667 29228159

[52] Pristipino C Beltrame JF Finocchiaro ML Hattori R Fujita M Mongiardo R Major racial differences in coronary constrictor response between Japanese and caucasians with recent myocardial infarction. *Circulation.* (2000) 101:1102–8. 10.1161/01.CIR.101.10.1102 10715255

[53] Sato K Kaikita K Nakayama N Horio E Yoshimura H Ono T Coronary vasomotor response to intracoronary acetylcholine injection, clinical features, and long-term prognosis in 873 consecutive patients with coronary spasm: analysis of a single-center study over 20 years. *J Am Heart Assoc.* (2013) 2:227. 10.1161/JAHA.113.000227 23858100 PMC3828805

[54] Nobuyoshi M Abe M Nosaka H Kimura T Yokoi H Hamasaki N Statistical analysis of clinical risk factors for coronary artery spasm: identification of the most important determinant. *Am Heart J.* (1992) 124:32–8. 10.1016/0002-8703(92)90917-K 1615825

[55] Ong P Athanasiadis A Borgulya G Voehringer M Sechtem U. 3-year follow-up of patients with coronary artery spasm as cause of acute coronary syndrome. *J Am Coll Cardiol.* (2011) 57:147–52. 10.1016/j.jacc.2010.08.626 21211685

[56] Lobo AS Cantu SM Sharkey SW Grey EZ Storey K Witt D Revascularization in patients with spontaneous coronary artery dissection and st-segment elevation myocardial infarction. *J Am Coll Cardiol.* (2019) 74:1290–300. 10.1016/j.jacc.2019.06.065 31488265

[57] Raphael CE Heit JA Reeder GS Bois MC Maleszewski JJ Tilbury RT Coronary embolus: an underappreciated cause of acute coronary syndromes. *JACC Cardiovasc Interv.* (2018) 11:172–80. 10.1016/j.jcin.2017.08.057 29348012

[58] Shibata T Kawakami S Noguchi T Tanaka T Asaumi Y Kanaya T Prevalence, clinical features, and prognosis of acute myocardial infarction attributable to coronary artery embolism. *Circulation.* (2015) 132:241–50. 10.1161/CIRCULATIONAHA.114.015134 26216084

[59] Scalone G Niccoli G Crea F. Editor’s choice- pathophysiology, diagnosis and management of MINOCA: an update. *Eur Heart J Acute Cardiovasc Care.* (2019) 8:54–62. 10.1177/2048872618782414 29952633

[60] Akashi YJ Nef HM Lyon AR. Epidemiology and pathophysiology of takotsubo syndrome. *Nat Rev Cardiol.* (2015) 12:387–97. 10.1038/nrcardio.2015.39 25855605

[61] Ghadri J-R Wittstein IS Prasad A Sharkey S Dote K Akashi YJ International expert consensus document on takotsubo syndrome (part II): diagnostic workup. Outcome, and management. *Eur Heart J.* (2018) 39:2047–62. 10.1093/eurheartj/ehy077 29850820 PMC5991205

[62] Ghadri J-R Wittstein IS Prasad A Sharkey S Dote K Akashi YJ International expert consensus document on takotsubo syndrome (part I): clinical characteristics. Diagnostic criteria, and pathophysiology. *Eur Heart J.* (2018) 39:2032–46. 10.1093/eurheartj/ehy076 29850871 PMC5991216

[63] Lyon AR Citro R Schneider B Morel O Ghadri JR Templin C Pathophysiology of takotsubo syndrome. *J Am Coll Cardiol.* (2021) 77:902–21. 10.1016/j.jacc.2020.10.060 33602474

[64] Pilgrim TM Wyss TR. Takotsubo cardiomyopathy or transient left ventricular apical ballooning syndrome: a systematic review. *Int J Cardiol.* (2008) 124:283–92. 10.1016/j.ijcard.2007.07.002 17651841

[65] Templin C Ghadri JR Diekmann J Napp LC Bataiosu DR Jaguszewski M Clinical features and outcomes of takotsubo (stress) cardiomyopathy. *New England J Med.* (2015) 373:929–38. 10.1056/NEJMoa1406761 26332547

[66] Tornvall P Collste O Ehrenborg E Järnbert-Petterson HA. Case-control study of risk markers and mortality in takotsubo stress cardiomyopathy. *J Am Coll Cardiol.* (2016) 67:1931–6. 10.1016/j.jacc.2016.02.029 27102508

[67] Stiermaier T Eitel C Desch S Fuernau G Schuler G Thiele H Incidence, determinants and prognostic relevance of cardiogenic shock in patients with Takotsubo cardiomyopathy. *Eur Heart J Acute Cardiovasc Care.* (2016) 5:489–96. 10.1177/2048872615612456 26474843

[68] Tschöpe C Ammirati E Bozkurt B Caforio ALP Cooper LT Felix SB Myocarditis and inflammatory cardiomyopathy: current evidence and future directions. *Nat Rev Cardiol.* (2021) 18:169–93. 10.1038/s41569-020-00435-x 33046850 PMC7548534

[69] Caforio ALP Pankuweit S Arbustini E Basso C Gimeno-Blanes J Felix SB Current state of knowledge on aetiology, diagnosis, management, and therapy of myocarditis: a position statement of the European society of cardiology working group on myocardial and pericardial diseases. *Eur Heart J.* (2013) 34:2636–48. 10.1093/eurheartj/eht210 23824828

[70] Ferreira VM Schulz-Menger J Holmvang G Kramer CM Carbone I Sechtem U Cardiovascular magnetic resonance in nonischemic myocardial inflammation. *J Am Coll Cardiol.* (2018) 72:3158–76. 10.1016/j.jacc.2018.09.072 30545455

[71] Cooper LT Baughman KL Feldman AM Frustaci A Jessup M Kuhl U The role of endomyocardial biopsy in the management of cardiovascular disease. *Circulation.* (2007) 116:2216–33. 10.1161/CIRCULATIONAHA.107.186093 17959655

[72] Tornvall P Gerbaud E Behaghel A Chopard R Collste O Laraudogoitia E Myocarditis or “true” infarction by cardiac magnetic resonance in patients with a clinical diagnosis of myocardial infarction without obstructive coronary disease: a meta-analysis of individual patient data. *Atherosclerosis.* (2015) 241:87–91. 10.1016/j.atherosclerosis.2015.04.816 25967935

[73] McKenna WJ Maron BJ Thiene G. Classification, epidemiology, and global burden of cardiomyopathies. *Circ Res.* (2017) 121:722–30. 10.1161/CIRCRESAHA.117.309711 28912179

[74] Dastidar AG Baritussio A de Garate E Drobni Z Biglino G Singhal P Prognostic role of CMR and conventional risk factors in myocardial infarction with nonobstructed coronary arteries. *JACC Cardiovasc Imaging.* (2019) 12:1973–82. 10.1016/j.jcmg.2018.12.023 30772224

[75] Gulati A Ismail TF Ali A Hsu L-Y Gonçalves C Ismail NA Microvascular dysfunction in dilated cardiomyopathy. *JACC Cardiovasc Imaging.* (2019) 12:1699–708. 10.1016/j.jcmg.2018.10.032 30660522 PMC8616858

[76] Crea F Montone RA Rinaldi R. Pathophysiology of coronary microvascular dysfunction. *Circ J.* (2021) 2021:848. 10.1253/circj.CJ-21-0848 34759123

[77] Mangiacapra F Viscusi MM Paolucci L Nusca A Melfi R Ussia GP The pivotal role of invasive functional assessment in patients with myocardial infarction with non-obstructive coronary arteries (MINOCA). *Front Cardiovasc Med.* (2021) 8:781485. 10.3389/fcvm.2021.781485 34869695 PMC8637881

[78] Mileva N Nagumo S Mizukami T Sonck J Berry C Gallinoro E Prevalence of coronary microvascular disease and coronary vasospasm in patients with nonobstructive coronary artery disease: systematic review and meta-analysis. *J Am Heart Assoc.* (2022) 11:3207. 10.1161/JAHA.121.023207 35301851 PMC9075440

[79] Mauricio R Srichai MB Axel L Hochman JS Reynolds HR. Stress cardiac MRI in women with myocardial infarction and nonobstructive coronary artery disease. *Clin Cardiol.* (2016) 39:596–602. 10.1002/clc.22571 27459149 PMC6490801

[80] Gudenkauf B Hays AG Tamis-Holland J Trost J Ambinder DI Wu KC Role of multimodality imaging in the assessment of myocardial infarction with nonobstructive coronary arteries: beyond conventional coronary angiography. *J Am Heart Assoc.* (2022) 11:2787. 10.1161/JAHA.121.022787 34970915 PMC9075186

[81] Montone RA Jang I-K Beltrame JF Sicari R Meucci MC Bode M The evolving role of cardiac imaging in patients with myocardial infarction and non-obstructive coronary arteries. *Prog Cardiovasc Dis.* (2021) 68:78–87. 10.1016/j.pcad.2021.08.004 34600948

[82] Pristipino C Sievert H D’Ascenzo F Louis Mas J Meier B Scacciatella P European position paper on the management of patients with patent foramen ovale. General approach and left circulation thromboembolism. *Eur Heart J.* (2019) 40:3182–95. 10.1093/eurheartj/ehy649 30358849

[83] Pathik B Raman B Mohd Amin NH Mahadavan D Rajendran S McGavigan AD Troponin-positive chest pain with unobstructed coronary arteries: incremental diagnostic value of cardiovascular magnetic resonance imaging. *Eur Heart J Cardiovasc Imaging.* (2016) 17:1146–52. 10.1093/ehjci/jev289 26590396

[84] Dastidar AG Rodrigues JCL Johnson TW de Garate E Singhal P Baritussio A Myocardial infarction with nonobstructed coronary arteries. *JACC Cardiovasc Imaging.* (2017) 10:1204–6. 10.1016/j.jcmg.2016.11.010 28109935

[85] Luis SA Luis CR Habibian M Lwin MT Gadowski TC Chan J Prognostic value of cardiac magnetic resonance imaging in acute coronary syndrome patients with troponin elevation and nonobstructive coronary arteries. *Mayo Clin Proc.* (2021) 96:1822–34. 10.1016/j.mayocp.2020.11.026 33992454

[86] Ananthakrishna R Liang Z Raman B Moran JL Rajvi B Patil S Long-term clinical outcomes in patients with a working diagnosis of myocardial infarction with non-obstructed coronary arteries (MINOCA) assessed by cardiovascular magnetic resonance imaging. *Int J Cardiol.* (2022) 349:12–7. 10.1016/j.ijcard.2021.11.088 34864074

[87] Sörensson P Ekenbäck C Lundin M Agewall S Bacsovics Brolin E Caidahl K Early comprehensive cardiovascular magnetic resonance imaging in patients with myocardial infarction with nonobstructive coronary arteries. *JACC Cardiovasc Imaging.* (2021) 14:1774–83. 10.1016/j.jcmg.2021.02.021 33865778

[88] Talebi S Moreno P Dominguez AC Tamis-Holland JE. The imaging toolbox to assess patients with suspected myocardial infarction in the absence of obstructive coronary artery disease (MINOCA). *Curr Cardiol Rep.* (2020) 22:134. 10.1007/s11886-020-01379-x 32910364

[89] Lintingre P-F Nivet H Clément-Guinaudeau S Camaioni C Sridi S Corneloup O High-resolution late gadolinium enhancement magnetic resonance for the diagnosis of myocardial infarction with nonobstructed coronary arteries. *JACC Cardiovasc Imaging.* (2020) 13:1135–48. 10.1016/j.jcmg.2019.11.020 31954658

[90] Zaya M Mehta PK Bairey Merz CN. Provocative testing for coronary reactivity and spasm. *J Am Coll Cardiol.* (2014) 63:103–9. 10.1016/j.jacc.2013.10.038 24201078 PMC3914306

[91] Takahashi T Samuels BA Li W Parikh MA Wei J Moses JW Safety of provocative testing with intracoronary acetylcholine and implications for standard protocols. *J Am Coll Cardiol.* (2022) 79:2367–78. 10.1016/j.jacc.2022.03.385 35710187 PMC8972358

[92] AlBadri A Bairey Merz CN Johnson BD Wei J Mehta PK Cook-Wiens G Impact of abnormal coronary reactivity on long-term clinical outcomes in women. *J Am Coll Cardiol.* (2019) 73:684–93. 10.1016/j.jacc.2018.11.040 30765035 PMC6383781

[93] Kelshiker MA Seligman H Howard JP Rahman H Foley M Nowbar AN Coronary flow reserve and cardiovascular outcomes: a systematic review and meta-analysis. *Eur Heart J.* (2022) 43:1582–93. 10.1093/EURHEARTJ/EHAB775 PMC9020988 34849697

[94] Machanahalli Balakrishna A Ismayl M Thandra A Walters R Ganesan V Anugula D Diagnostic value of cardiac magnetic resonance imaging and intracoronary optical coherence tomography in patients with a working diagnosis of myocardial infarction with non-obstructive coronary arteries – a systematic review and meta-analysis. *Curr Probl Cardiol.* (2022) 2022:101126. 10.1016/j.cpcardiol.2022.101126 35120967

[95] Pelliccia F Pepine CJ Berry C Camici PG. The role of a comprehensive two-step diagnostic evaluation to unravel the pathophysiology of MINOCA: a review. *Int J Cardiol.* (2021) 336:1–7. 10.1016/j.ijcard.2021.05.045 34087335

[96] Mukherjee D. Myocardial infarction with nonobstructive coronary arteries: a call for individualized treatment. *J Am Heart Assoc.* (2019) 8:e013361. 10.1161/JAHA.119.013361 31284819 PMC6662152

[97] Xing L Yamamoto E Sugiyama T Jia H Ma L Hu S EROSION study (effective anti-thrombotic therapy without stenting: intravascular optical coherence tomography–based management in plaque erosion). *Circ Cardiovasc Interv.* (2017) 10:5860. 10.1161/CIRCINTERVENTIONS.117.005860 29246916

[98] Slavich M Patel RS. Coronary artery spasm: current knowledge and residual uncertainties. *IJC Heart Vasculat.* (2016) 10:47–53. 10.1016/j.ijcha.2016.01.003 28616515 PMC5462634

[99] Ortega-Paz L Galli M Capodanno D Brugaletta S Angiolillo DJ. The role of antiplatelet therapy in patients with MINOCA. *Front Cardiovasc Med.* (2022) 8:1297. 10.3389/fcvm.2021.821297 35237672 PMC8882905

[100] Cerrato E Giacobbe F Quadri G Macaya F Bianco M Mori R Antiplatelet therapy in patients with conservatively managed spontaneous coronary artery dissection from the multicentre DISCO registry. *Eur Heart J.* (2021) 42:3161–71. 10.1093/eurheartj/ehab372 34338759

[101] Marinescu MA Löffler AI Ouellette M Smith L Kramer CM Bourque JM. Coronary microvascular dysfunction, microvascular angina, and treatment strategies. *JACC Cardiovasc Imaging.* (2015) 8:210–20. 10.1016/J.JCMG.2014.12.008 25677893 PMC4384521

[102] Zilio F Muraglia S Bonmassari R. Cardiac arrest complicating cardiogenic shock: from pathophysiological insights to impella-assisted cardiopulmonary resuscitation in a pheochromocytoma-induced takotsubo cardiomyopathy—a case report. *Eur Heart J Case Rep.* (2021) 5:92. 10.1093/ehjcr/ytab092 34113770 PMC8186919

[103] Ramanath VS Armstrong DF Grzybowski M Rahnama-Mohagdam S Tamhane UU Gordon K Receipt of cardiac medications upon discharge among men and women with acute coronary syndrome and nonobstructive coronary artery disease. *Clin Cardiol.* (2010) 33:36–41. 10.1002/clc.20701 20063300 PMC2935808

[104] Maddox TM Ho PM Roe M Dai D Tsai TT Rumsfeld JS. Utilization of secondary prevention therapies in patients with nonobstructive coronary artery disease identified during cardiac catheterization. *Circ Cardiovasc Qual Outcomes.* (2010) 3:632–41. 10.1161/CIRCOUTCOMES.109.906214 20923997

[105] Pitts R Daugherty SL Tang F Jones P Ho PM Tsai TT Optimal secondary prevention medication use in acute myocardial infarction patients with nonobstructive coronary artery disease is modified by management strategy: insights from the TRIUMPH Registry. *Clin Cardiol.* (2017) 40:347–55. 10.1002/clc.22686 28387960 PMC5596450

[106] Smilowitz NR Dubner R Hellkamp AS Widmer RJ Reynolds HR. Variability of discharge medical therapy for secondary prevention among patients with myocardial infarction with non-obstructive coronary arteries (MINOCA) in the united states. *PLoS One.* (2021) 16:e0255462. 10.1371/journal.pone.0255462 34339469 PMC8328325

[107] Masson W Lobo M Barbagelata L Lavalle-Cobo A Molinero G. Prognostic value of statin therapy in patients with myocardial infarction with nonobstructive coronary arteries (MINOCA): a meta-analysis. *Acta Cardiol.* (2021) 2021:1–8. 10.1080/00015385.2021.1955480 34308792

[108] Ciliberti G Verdoia M Merlo M Zilio F Vatrano M Bianco F Pharmacological therapy for the prevention of cardiovascular events in patients with myocardial infarction with non-obstructed coronary arteries (MINOCA): insights from a multicentre national registry. *Int J Cardiol.* (2021) 327:9–14. 10.1016/j.ijcard.2020.11.040 33242505

[109] Bossard M Gao P Boden W Steg G Tanguay J-F Joyner C Antiplatelet therapy in patients with myocardial infarction without obstructive coronary artery disease. *Heart.* (2021) 107:1739–47. 10.1136/heartjnl-2020-318045 33504513

[110] Nordenskjöld AM Agewall S Atar D Baron T Beltrame J Bergström O Randomized evaluation of beta blocker and ACE-inhibitor/angiotensin receptor blocker treatment in patients with myocardial infarction with non-obstructive coronary arteries (MINOCA-BAT): rationale and design. *Am Heart J.* (2021) 231:96–104. 10.1016/j.ahj.2020.10.059 33203618

[111] Serpytis R Majauskiene E Navickas P Lizaitis M Glaveckaite S Rucinskas K Randomized pilot trial on optimal treatment strategy, myocardial changes, and prognosis of patients with myocardial infarction with nonobstructive coronary arteries (MINOCA). *Am J Med.* (2022) 135:103–9. 10.1016/j.amjmed.2021.08.023 34562410

[112] Montone RA Cosentino N Graziani F Gorla R del Buono MG la Vecchia G Precision medicine versus standard of care for patients with myocardial infarction with non-obstructive coronary arteries (MINOCA): rationale and design of the multicentre, randomised PROMISE trial. *EuroIntervention.* (2022) 2022:178. 10.4244/EIJ-D-22-00178 35734824 PMC9743237

